# Editorial: Advances in pluripotent stem cell-based *in vitro* models of the human heart for cardiac physiology, disease modeling and clinical applications

**DOI:** 10.3389/fphys.2024.1378495

**Published:** 2024-02-19

**Authors:** Maksymilian Prondzynski, Josè Manuel Pioner, Luca Sala, Milena Bellin, Viviana Meraviglia

**Affiliations:** ^1^ Department of Cardiology, Boston Children’s Hospital, Boston, MA, United States; ^2^ Harvard Medical School, Boston, MA, United States; ^3^ Department of Biology, University of Florence, Florence, Italy; ^4^ Istituto Auxologico Italiano IRCCS, Center for Cardiac Arrhythmias of Genetic Origin and Laboratory of Cardiovascular Genetics, Milan, Italy; ^5^ Department of Biotechnology and Biosciences, University of Milano-Bicocca, Milan, Italy; ^6^ Department of Anatomy and Embryology, Leiden University Medical Center, Leiden, Netherlands; ^7^ Department of Biology, University of Padua, Padua, Italy; ^8^ Veneto Institute of Molecular Medicine, Padua, Italy

**Keywords:** human induced pluripotent stem cell (hiPSC), hiPSC-derived cardiac cells, multicellular 3D cardiac models, disease modeling and treatment, human cardiac pathophysiology

Cardiovascular diseases are the leading cause of mortality in developed countries. The generation and validation of reliable *in vitro* models that closely mimic the human heart is crucial to enhance our knowledge of the molecular mechanisms underlying cardiac physiology in health and disease. The advent of human induced pluripotent stem cells (hiPSCs) and their ability to differentiate into cardiovascular cells generated new opportunities for disease modeling, drug discovery, and personalized/regenerative medicine. Recent progresses in cell biology and tissue engineering enabled the generation of advanced *in vitro* tools capturing physiological and pathological properties of the heart. This has the potential to shed new light on innovative strategies for treating cardiac diseases. Yet, there is still a continuous need to increase the level of complexity for *in vitro* cardiac models with enhanced (patho)physiological relevance and mimicry of the native human heart.

As editors of this Research Topic, we reviewed with great interest a collection of seven manuscripts (two reviews, four original research articles and one commentary) and here we will summarize the key findings of the published articles.

Up to date, many different advanced *in vitro* tools modeling cardiomyocyte and/or non-cardiomyocyte function, and specific aspects of the human heart, have been developed and are summarized in the review by Vuorenpää et al. The authors discussed future directions and opportunities of more complex *in vitro* micro-physiological systems and their impact on modeling the (patho)physiology of human cardiac function, highlighting the importance of monitoring the microenvironment as well as the biological complexity in currently utilized and future *in vitro* tissues.

Next to understanding the impact of genetic conditions on cardiomyocyte function, it becomes more evident that non-cardiomyocytes can also affect disease states in the heart. A mini-review by Rabino et al. summarizes studies focused on investigating hiPSC-derived endothelial cell function in the context of inherited cardiomyopathies. The review highlights not only the impact of dysfunctional endothelium in the development and progression of genetic cardiomyopathies, but also new research directions and potential targets exploitable for therapeutic intervention.

In the study by Monnerat et al. the authors characterized hiPSC-derived cardiomyocytes (hiPSC-CMs) from a patient affected by Hutchinson-Gilford Progeria Syndrome (HGPS) to model the cardiac defects associated with this rare genetic disorder. To this purpose, they applied a series of morpho-functional, biophysical and molecular approaches and broadly outlined the phenotypic features characterizing HGPS hiPSC-CMs. Specifically, HGPS hiPSC-CMs showed altered nuclear morphology and abnormal mitochondrial structures in terms of decreased mitochondrial volume and lower number of cristae per mitochondrion. Proteomics analysis revealed alterations in key metabolic processes such as amino-acid biosynthesis, cellular stress response and citric acid cycle. This study provides novel results to understand the processes underlying the accelerated cardiac aging associated with HGPS and induced by morphological and biochemical alterations, highlighting the potential relation between premature cardiac aging and mitochondrial dysfunctions.

The article by Benzoni et al. investigated a *PTIX2* gain of function mutation associated with the development of atrial fibrillation (AF). Specifically, atrial hiPSC-CMs from a patient-specific line and its isogenic control were used to investigate mitochondrial dysfunctions downstream to PITX2 alterations, revealing a higher mitochondrial content, activity and superoxide production in basal conditions. Interestingly, the stimulation of mitochondrial activity further exposed a deficiency in ATP production, highlighting a possible link between mitochondrial defects and oxidative stress as triggers for arrhythmogenesis. Importantly, this research emphasizes that the understanding of common mechanisms predisposing to genetic AF might increase our knowledge on processes underlying multifactorial forms of AF.

The study by Pioner et al. offers a detailed perspective on the time-dependent maturation and substrate-stiffness-dependent changes of calcium homeostasis in hiPSC-CMs from Duchenne Muscular Dystrophy (DMD). The study demonstrates that DMD hiPSC-CMs have reduced calcium transient amplitude and slower kinetics when compared to their gene-edited isogenic controls, with differences in calcium transient amplitude getting wider along with the maturation time. Also, DMD hiPSC-CMs lose the ability to adapt their calcium homeostasis when cultured on stiffer substrates intended to resemble the conditions of myocardial fibrosis. These findings provide a better understanding on how the cardiomyopathy phenotype develops and progresses in DMD. This study has important implications; firstly, it demonstrates that maturation strategies have become essential for disease modeling of multifactorial diseases, providing insights into the crucial role of substrate stiffness to model muscular dystrophies and cardiomyopathies. Secondly, it reinforces the importance of well-calibrated and executed functional studies to capture complex aspects of disease progression.

The advancement of *in vitro* cardiac models not only relies on a better understanding of cardiac function and disease mechanisms, but also on more representative human *in vitro* platforms that might be useful for clinical applications as addressed in the article by Feaster et al. The authors presented a tridimensional (3D) human engineered cardiac tissues (ECTs) as a model to investigate the impact of cardiac contractility modulation (CCM), a promising medical device therapy for heart failure with reduced ejection fraction. The authors verified a robust contractile response of 3D ECTs to CCM when stimulated by a range of electrical pulses (“doses”), while this response in conventional bidimensional (2D) hiPSC-CMs monolayers remained unaffected. Specifically, 3D ECTs displayed an increased force and accelerated kinetics under acute CCM stimulation in a pulse-dependent manner.

The value of 3D *in vitro* models to evaluate safety and efficacy of novel cardiac devices, including CCM, is supported by a commentary on this article by Bierhuizen et al. Here, the authors emphasized the need of pre-clinical CCM testing in human tissues to better understand the underlying CCM mechanisms. In support of patient-specific models, they proposed similar micro-physiological systems applied to human living myocardial slices (LMS) as an additional *in vitro* platform for CCM testing.

Taken together, the contributions published within this Research Topic collect an overview of the state-of-the-art *in vitro* human cardiac systems to improve our understanding of cardiac physiology and diseases, providing more efficient tools for therapy development and personalized medicine. This collection also provides insights into future directions to develop more accurate models that best recapitulate the (patho)physiology of the human native tissue.

